# Systematic Down-Selection of Repurposed Drug Candidates for COVID-19

**DOI:** 10.3390/ijms231911851

**Published:** 2022-10-06

**Authors:** Christopher A. MacRaild, Muzaffar-Ur-Rehman Mohammed, Faheem, Sankaranarayanan Murugesan, Ian K. Styles, Amanda L. Peterson, Carl M. J. Kirkpatrick, Matthew A. Cooper, Enzo A. Palombo, Moana M. Simpson, Hardik A. Jain, Vinti Agarwal, Alexander J. McAuley, Anupama Kumar, Darren J. Creek, Natalie L. Trevaskis, Seshadri S. Vasan

**Affiliations:** 1 Drug Delivery, Disposition and Dynamics, Monash Institute of Pharmaceutical Sciences, Monash University, Parkville, VIC 3800, Australia; 2 Department of Pharmacy, Birla Institute of Technology and Science, Pilani Campus, Pilani 333031, Rajasthan, India; 3 Department of Medicinal Chemistry, University of Utah, Salt Lake City, UT 84112, USA; 4 Bio21 Institute, University of Melbourne, Parkville, VIC 3052, Australia; 5 Centre for Medicine Use and Safety, Monash Institute of Pharmaceutical Sciences, Monash University, Parkville, VIC 3800, Australia; 6 Institute for Molecular Bioscience, The University of Queensland, Brisbane, QLD 4072, Australia; 7 Department of Chemistry and Biotechnology, Swinburne University of Technology, Hawthorn, VIC 3122, Australia; 8 Griffith Institute for Drug Discovery, Griffith University, Nathan, QLD 4111, Australia; 9 Department of Electrical and Electronics Engineering, Birla Institute of Technology and Science, Pilani 333031, Rajasthan, India; 10 Department of Computer Science and Information Systems, Birla Institute of Technology and Science, Pilani 333031, Rajasthan, India; 11 Commonwealth Scientific and Industrial Research Organisation, Australian Centre for Disease Preparedness, Portarlington Road, Geelong, VIC 3220, Australia; 12 Commonwealth Scientific and Industrial Research Organisation, Land and Water, Waite Campus, SA 5064, Australia; 13 Department of Health, 189 Royal Street, East Perth, WA 6004, Australia; 14 Department of Health Sciences, University of York, York YO10 5DD, UK

**Keywords:** COVID-19, CoviRx.org, database, drugs, pandemic, repurposing, SARS-CoV-2, therapies, treatments, Variants of Concern (VOC)

## Abstract

SARS-CoV-2 is the cause of the COVID-19 pandemic which has claimed more than 6.5 million lives worldwide, devastating the economy and overwhelming healthcare systems globally. The development of new drug molecules and vaccines has played a critical role in managing the pandemic; however, new variants of concern still pose a significant threat as the current vaccines cannot prevent all infections. This situation calls for the collaboration of biomedical scientists and healthcare workers across the world. Repurposing approved drugs is an effective way of fast-tracking new treatments for recently emerged diseases. To this end, we have assembled and curated a database consisting of 7817 compounds from the Compounds Australia Open Drug collection. We developed a set of eight filters based on indicators of efficacy and safety that were applied sequentially to down-select drugs that showed promise for drug repurposing efforts against SARS-CoV-2. Considerable effort was made to evaluate approximately 14,000 assay data points for SARS-CoV-2 FDA/TGA-approved drugs and provide an average activity score for 3539 compounds. The filtering process identified 12 FDA-approved molecules with established safety profiles that have plausible mechanisms for treating COVID-19 disease. The methodology developed in our study provides a template for prioritising drug candidates that can be repurposed for the safe, efficacious, and cost-effective treatment of COVID-19, long COVID, or any other future disease. We present our database in an easy-to-use interactive interface (CoviRx that was also developed to enable the scientific community to access to the data of over 7000 potential drugs and to implement alternative prioritisation and down-selection strategies.

## 1. Introduction

COVID-19 is the most devastating natural calamity of the 21st century. It emerged in December 2019 and in March 2020 was declared a pandemic by World Health Organisation (WHO) [1]. Since the beginning of the pandemic, SARS-CoV-2 has infected over 620 million people and has claimed more than 6.5 million lives across the world, and up to 26.7 million excess deaths were reported as of September 2022 [2,3]. Most of these deaths have resulted from the complications of the SARS-CoV-2 infection, such as systemic inflammatory response syndrome (SIRS); organ dysfunction and failure (particularly acute respiratory and renal failure); and thrombotic, neurological, and cardiovascular complications [4,5,6]. A significant proportion of patients also endure symptoms long after the acute viral infection phase has passed, so-called “long covid” [7]. The emergency of the situation has led to extraordinary efforts from health care workers and biomedical scientists across the globe to seek approaches to mitigate the severity and impact of the disease. A range of vaccines using different technologies such as viral vector, RNA, and protein subunit vaccines are now approved for use and have rolled out worldwide [8,9,10]. These vaccines have successfully reduced the risk of transmission, the severity of the disease, and hospitalisations [9]. However, none of the vaccines have proven to be entirely effective at blocking infection of the virus, and breakthrough infections that lead to severe disease remain [11]. The available vaccines are also contraindicated or less effective in specific patient populations, such as the immunocompromised. Therefore, we need to continue to seek additional solutions to reduce the health, and consequently societal, impacts of COVID-19.

Several antiviral drugs have been repurposed or developed to reduce SARS-CoV-2 replication, including small molecule antivirals remdesivir, molnupiravir, and nirmatrelvir (in combination with ritonavir to extend plasma residence time) [12]. Currently, remdesivir is the only drug molecule that has received approval from the FDA, although its intravenous route of administration has dramatically diminished its application. While molnupiravir was initially shown to be effective in reducing hospitalisation or death by almost 50%, the final analysis revealed that molnupiravir was only 30% effective in the MOVe-OUT study [13]. Moreover, there are some concerns over its potential mutagenicity to the host [14]. While ritonavir-boosted nirmatrelvir (paxlovid) shows great promise in reducing hospitalisation or death, it is prone to complex drug–drug interactions due to the ritonavir component of paxlovid [15]. Four monoclonal antibodies directed toward epitopes of the spike protein receptor-binding domain (RBD) of SARS-CoV-2 have received emergency use authorisation from the US Food and Drug Administration (FDA): bamlanivimab plus etesevimab, casirivimab plus imdevimab, sotrovimab, and tixagevimab plus cilgavimab. There is concern that these monoclonal antibodies will have reduced activity against future variants with different spike protein structures or none at all. Indeed, bamlanivimab plus etesevimab and casirivimab plus imdevimab have already been paused by the FDA due to reduced activity against the omicron variant [16,17]. The systemic inflammatory response to the virus is also being treated using immunomodulatory drugs that include corticosteroids, monoclonal antibodies, IL-1 receptor antagonists, and Janus kinase inhibitors [18,19].

Hence, there is a prescient need to identify further effective treatments to neutralise the virus, which may be used alone or in combination with the currently available therapies. A promising strategy to find potential COVID-19 treatments is to repurpose drugs that are already approved for other diseases by major regulators and have established and proven safety profiles. This can reduce the cost and time of drug development. Indeed, a range of studies have evaluated the potential antiviral effects of compounds in high throughput screening assays. Some of these drugs have progressed to clinical trials, with promising results for the common anti-depressant, fluvoxamine. Whilst fluvoxamine reduced hospitalisation by ~30%, there remains room to find more effective drug treatments. From this starting point, our ‘Drug Selection Committee (DSC)’, comprising experts across a range of relevant disciplines, assembled a database of drugs for repurposing against SARS-CoV-2. Together, we decided on specific criteria to down-select the most promising drug candidates. The methodology we developed provides a template for prioritising drug candidates that are safe, efficacious, and cost-effective for COVID-19 and that may be useful in tackling future diseases. To share the findings of our efforts with the world, we also created an online database (CoviRx, https://www.covirx.org/ (accessed on 3 October 2022)) which is described in a matching publication [20]. Herein, we focus on the process used to down-select the top drug candidates, present our ‘top 12′ candidates, and analyse the potential mechanisms of these drugs.

## 2. Results

### 2.1. Assembly of the Data

**Figure 1:**
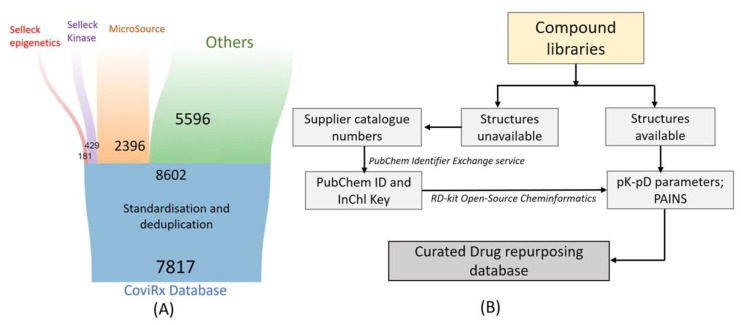
(**A**) Assembly of the CoviRx database, showing compound numbers from the original commercial sources (**top**) flowing into the Open Drug collection (**middle**) and though to the final database (**bottom**) (**B**) Workflow of the data curation process.

Due to the lack of a specific database for drug repurposing against SARS-CoV-2, we assembled a database that is publicly available as CoviRx (https://www.covirx.org/ (accessed on 3 October 2022)). We took the Compounds Australia Open Drug collection as our starting point to ensure the ready availability of selected compounds in assay-ready formats [21]. This collection comprises of ~8000 compounds derived from several commercial collections (Figure 1). Many of these compounds have regulatory approval from the U.S. FDA or Australian Therapeutics Goods Administration (TGA), while others are under clinical investigation or are approved in other jurisdictions. Metadata covering compounds’ physicochemical properties and biological data such as the target and pathways, mechanism of action, IC_50_ values for the original clinical indication, pharmacokinetics, safety, etc., were extracted from a range of online sources, as detailed in Table 1. After data standardisation and the removal of duplicate entries (see Methods), we were left with a final dataset of 7817 compounds (Figure 1). From here, the results of nine in vitro antiviral assays were mapped to the database [22,23,24,25,26,27,28,29]. These assays are summarised in Section 2.3.

### 2.2. The CoviRx Database Reveals Host-Cell Pathways

**Figure 2:**
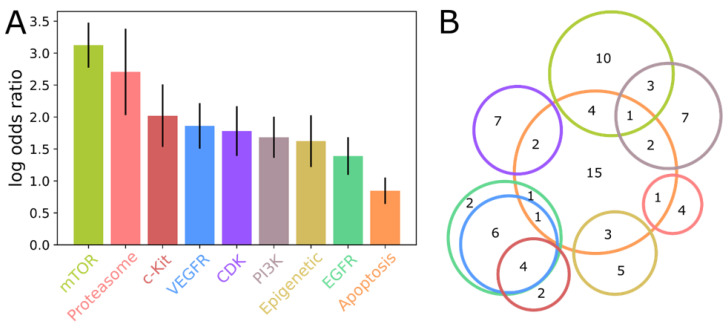
Enrichment analysis of molecular targets associated with SARS-CoV-2 activity. (**A**) The extent of enrichment, measured as log odds ratio, for nine targets significantly enriched in SARS-CoV-2 active compounds. (**B**) Venn diagram illustrating the degree of overlap amongst enriched targets. The colour scheme is shown in (**A**), and the number of active compounds contributing to each target combination is also shown.

To assess whether drugs targeting specific pathways or molecular targets are more likely to show activity against SARS-CoV-2, an enrichment analysis was performed on words describing the original target of each active compound in the CoviRx database. After multiple-testing correction to control the false discovery rate at α < 0.05, a total of nine informative terms were found to be significantly overrepresented amongst SARS-CoV-2 inhibiting compounds (Figure 2). It is striking that none of these terms were suggestive of a viral target; instead, they appear to point to host-cell pathways that the virus depends upon for survival and replication. This is perhaps consistent with the general lack of success in repurposing existing antiviral drugs against COVID-19 [47,48]. 

Examination of the individual active compounds associated with each enriched term highlights substantial overlap, implying that only a few host-cell pathways contribute to the signal identified here. In particular, four of the thirteen Phosphoinositide 3-kinases (PI3K) inhibitors that show SARS-CoV-2 activity are annotated as targeting the mammalian target of rapamycin (mTOR). Likewise, there is extensive overlap between the active compounds targeting the growth factor receptor kinases c-Kit, Vascular endothelial growth factor receptor (VEGFR), and endothelial growth factor receptor (EGFR). The enrichment of the apoptosis term comprises contributions from the growth factor receptor kinases, PI3K/mTOR axis, cyclin-dependent kinase (CDK) inhibitors, and from epigenetic modulators, possibly suggestive of a common mechanism linking all these diverse molecular targets. 

### 2.3. Down-Selection of Drugs Based on Available Data and Regulatory Approval Status

**Figure 3:**
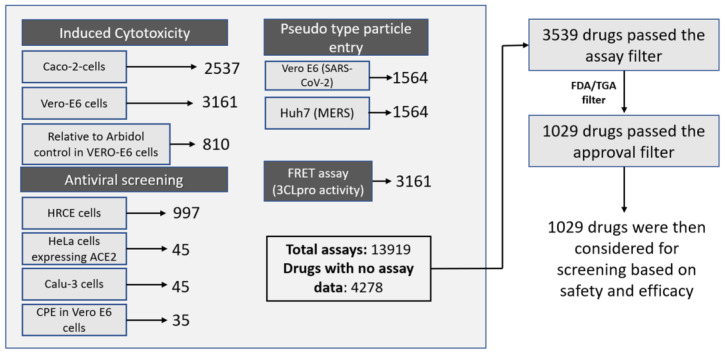
Antiviral assay data and number of drugs that passed each filter.

Using an iterative approach, the CoviRx database was subjected to a series of filtering criteria to identify those with potential for repurposing against SARS-CoV-2. The first filter sought to identify compounds for which at least one in vitro assay result was present [22,23,24,25,26,27,28,29]. The assays are summarised in Figure 3. The total number of drugs that failed this filter was 4278, and these compounds were excluded for further analysis. The next filter applied was the removal of drugs that are not approved by major regulatory bodies, as this would help identify compounds with a faster regulatory acceptance pathway. About 57% of the compounds were approved by the U.S. FDA (39%) and/or Australia (18%)**.** Additionally, investigational drug molecules were excluded from our study as these would require additional time to develop, including complete pharmacokinetic, safety, and pharmacological profiling [49]. Figure 3 summarises the types of assays found and the number of drugs that passed these first two filters. The remaining drugs were taken forward as input for implementing other filters based on the likely efficacy and safety of the compounds.

### 2.4. Down-Selection of Drugs Based on Indicators of Efficacy and Safety

**Figure 4:**
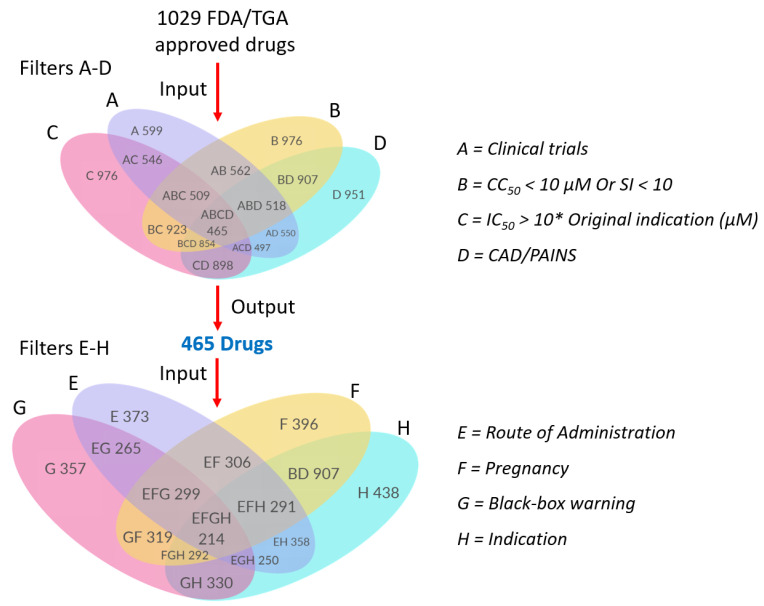
Down-selection of drugs based on indicators of efficacy and safety (A–D and E–H); * = folds.

Firstly, the drugs were down-selected based on their potential to provide a new treatment with in vivo efficacy against the virus (Figure 4, Flower 1). Each sub-filter and their need for implementation is discussed in more detail under the Methods section. To ensure that we were identifying new treatments and to avoid duplicating previous work, in Filter A, we removed drugs from drug classes that had already been tested in COVID-19 clinical trials as antiviral treatments, resulting in 599 remaining drugs. Filters B-D removed drugs that were unlikely to be efficacious and/or safe in vivo based on their IC_50_ against COVID-19 being >10* the original indication, selectivity index (CC_50_/IC_50_), and drugs likely to provide false-positive results in vitro (CAD/PAINS filter) [50,51,52,53]. A subset of 465 drugs passed all these filters. Next, the drugs were further down-selected based on potential safety and ease of use. We selected drugs that are administered via oral or inhalation routes as this would enable easy use at home (Filter E), those that are reasonably safe in pregnancy (Filter F), that carried no black box warnings (Filter G), and we removed any remaining drugs with unusual indications or that were diagnostics (Filter H). This sequential down-selection resulted in 214 drug molecules (Figure 4, Filters E–H).

## 3. Discussion

Following the application of robust filtering methodology to the drugs in the curated database, the final output was 214 potential drug candidates for COVID-19. Once these top 214 drugs were in place, they were sorted from those most likely to least likely to show antiviral activity based on their computed activity rank score. This resulted in the top 15 candidates being selected (Table 2). The team then evaluated the top 15 drugs in greater detail to determine promising candidates for repurposing. The top 15 drugs, along with their rank score, original indication, mechanism of action, and associated targets, are represented in Table 2. It is interesting to know that the drugs obtained in this list are from our down-selection methodology, and no attempts were made to select drugs based on their mechanisms.

Next, we examined each compound thoroughly to determine their likelihood of providing safe and effective treatments. We also decided to prioritise the evaluation of drugs belonging to diverse pharmacological classes. We noted that several histamine receptor antagonists were in the top list of drugs, so we evaluated the potential efficacy and safety profiles of all antihistamine drugs. This led to the selection of meclizine for further use. Moxidectin is a closely related analogue of ivermectin that has been extensively debated and tested as a potential drug for COVID-19. The results suggest it has poor IC_50_ against SARA-CoV-2 compared to ivermectin. Hence, it was not considered in further studies [54,55,56,57]. Deflazacort, a corticosteroid, was left out, as several corticosteroids are already in clinical trials and are being used clinically due to their anti-inflammatory properties in treating COVID-19 complications [58,59]. Cefaclor and other cephalosporin antibiotics were strongly considered but ultimately not included. Cefaclor performed the worst among a series of antibiotics against SARS-CoV-2 in the drug screening. Other cephalosporins were only active in in vitro antiviral assays at concentrations expected to be unachievable in in vivo screening [60]. There was insufficient literature available to consider nifurtimox for repurposing against COVID-19. Several anticholinergic drugs were near the top of the list, including procyclidine and tolterodine. These were considered undesirable due to anticholinergic side effects, which may be troublesome, particularly in the elderly. In addition, the drug concentrations required to inhibit the virus were high compared to plasma concentrations when used for their current therapeutic indication. Though cysteamine has some activity against SARS-CoV-2 in vitro [61,62], the concentration at which it exhibits its antiviral activity is too high, and attaining similar plasma concentrations in human patients would be difficult and likely result in off-target toxicities. Selective serotonin reuptake inhibitors (SSRI’s) are noteworthy to study due to their immunomodulatory effects, and their action against SARS-CoV-2 as fluvoxamine have shown good inhibition in tissue models [63]. As dapoxetine and mianserine belong to a similar category of drugs, these would prevent the cytokine storm, thereby preventing COVID-19-associated symptoms. In addition, rilpivirine is an antiretroviral drug. Hence, further evaluation of these drugs against SARS-CoV-2 should be considered.

Additionally, drugs that were filtered out but had an activity rank score < 0.2 were further investigated to see if they could be reconsidered for repurposing despite their shortcomings. Drugs that were reconsidered and their reason for initial removal are highlighted in Table 3. We reviewed each drug individually, and a decision was made not to pursue any of the drugs except mTOR inhibitors and NK-1 receptor antagonist (rolapitant). Among the mTOR’s, only everolimus was chosen, as zotarolimus is used in stents while pimecrolimus is administered topically. Although everolimus has a black box warning, this is only for skin cancer seen after long-term administration as an immunosuppressant. A short course of everolimus for COVID-19 may not cause such harmful effects to patients [64]. Additionally, there are convincing reasons available to repurpose mTOR inhibitors for use in COVID-19 due to their immunomodulatory and antiviral properties [64,65,66,67,68]. Similar arguments could also be made for rolapitant, a neurokinin-1 receptor (NK-1) antagonist. Substance P and NK-1 receptors have been hypothesised to play an aberrant role in the cytokine storm observed in COVID-19 patients [69,70,71]. A report published in a preprint server claims that aprepitant, a congener of rolapitant, has beneficial effects in COVID-19 patients when combined with dexamethasone [70]. Additionally, a clinical case report disclosed positive effects of aprepitant in a patient with post-acute COVID-19 syndrome [72]. These results encouraged us to examine further if rolapitant has antiviral activity against SARS-CoV-2. 

For the final prioritisation of the drugs for repurposing, we sought to determine whether the concentration required for an antiviral effect would be achievable in plasma after administration of typical therapeutic doses. We collected the single-dose maximum plasma concentration (Cmax) and average steady-state plasma concentration (Cssave) values obtained after administration of the current clinical doses. We then adjusted those values for each drug based on available plasma protein binding ratios (Table 4). Another consideration we made in selecting the final list was to look for other drugs belonging to the same class as serotonin receptor antagonists, antihistamines, and tyrosine kinase inhibitors (TKIs). These classes of drugs were ranked in the top 15. Accordingly, ondansetron (antiemetic), cyclizine (antihistamine), cetirizine (antihistamine), and lapatinib (tyrosine kinase inhibitor) were included from these classes for final consideration. Once the free drug concentration calculations were done for all 12 drugs, we calculated the free plasma drug concentrations that could effectively inhibit the virus [50,73,74]. The top 12 drugs listed in Table 4 were selected for final screening in relevant ex vivo models of COVID-19.

## 4. Materials and Methods

### 4.1. Database Assembly, Data Collection and Processing

The database was assembled by first extracting compound structures and all available metadata from the original supplier’s documentation. Where chemical structures were not available from the suppliers’ documentation, supplier catalogue numbers were converted to PubChem Compound IDs and InChiKey using the PubChem Identifier Exchange Service. ChEMBL, DrugBank, and PubChem identifiers were derived by the InChiKey lookup of the respective databases. Physicochemical properties were calculated using RDKit. RDKit was also used to identify pan-assay interference compounds based on established substructure filters [75]. Duplicate entries were identified based on unsuitable indication compound names or InChiKey and merged to a single consolidated entry. This resulted in a final set of 7817 compounds.

### 4.2. SARS-CoV-2 Assay Data Collection and Activity Rank Score Calculation

All the available SARS-CoV-2 assay data of the FDA/TGA approved drugs were extracted using the drug identifiers from ChEMBL and other published datasets (Figure 2) [22,23,24,25,26,27,28,29]. The 3539 drugs with 13,919 assay datasets were ranked for activity. These ranks were then converted to fractional ranks by dividing the total number of assay results for each assay in our dataset. The assay rank score for each compound was the mean of these fractional ranks’ overall assays in which the compound was tested. Thus, the assay score for compound *j* was given by:

$$A_{j}=\frac{1}{N_{j}}\sum_{i=1}^{N_{j}}\frac{r_{i,j}}{n_{i}}$$

where *i* runs over the *N_j_* assays in which compound *j* was assessed, *r*_*i*,*j*_ is the activity rank of compound *j* in assay *i*, and *n_i_* is the number of compounds in the CoviRx database assessed in assay *i*.

### 4.3. Filtration Methodology

Drugs have various pharmacological properties and sometimes result in severe life-threatening effects. In addition, various chemical compounds either lack therapeutic action or are inactive. Apart from these, there are possibilities to get false-positive results due to cationic and amphiphilic compounds (CAD) or pan-assay interference (PAINS). Hence, to obtain active drugs that are safe to use in healthy humans and pregnant women, a series of filters were used to further screen the drugs. All the filters and the reasons we used them are described briefly in Table 5.

### 4.4. Enrichment Analysis

The target enrichment analysis was performed by first assembling a list of all words appearing in the original target annotation for the compounds in the CoviRx database for which SARS-CoV-2 assay data are available. This list was edited manually to remove non-specific terms that were not informative of the molecular target, resulting in a list of 441 target terms. For each term, we constructed a contingency table of active and inactive compounds that are and are not annotated to target the specified term, where we define active compounds as those with an assay rank score less than 0.1. Enrichment was assessed with the Fisher exact test and resulting *p*-values were adjusted by the Benjamini-Hochberg procedure to control the false discovery rate to α < 0.05.

## 5. Conclusions

COVID-19 continues to cause havoc across most parts of the world [3,78]. While vaccines have significantly reduced disease severity and mortality, vaccine hesitancy, poor coverage, and a lack of efficacy against emerging variants of concern (VOCs) necessitate developing alternate clinical tools to deal with the virus and its future VOCs. There is an urgent and critical need to identify or develop drugs that can perturb viral replication in target tissues and reduce or prevent long-term health complications. Novel drug discovery and development suffer from certain drawbacks such as a high attrition rate and the significant time required to progress a drug into the market. At the same time, repurposing approved drugs provides an opportunity to circumvent such drawbacks and speed up the development process. Repurposed drugs could be expedited to phase 2–3 trials for COVID-19 if robust pre-clinical data are available. In the present study, an effort was made to assemble a database of more than 7000 compounds and systematically down-select potential drugs for repurposing against COVID-19. The down-selection was achieved by employing several filters that assessed various attributes required for the drug to be safe and effective, such as SARS-CoV-2 antiviral activity, approval status, PK–PD data, and toxicity. Drugs such as L-cycloserine, everolimus, pyrimethamine, cyclizine, lapatinib, and rolapitant were identified and prioritised for repurposing against COVID-19. The top 12 repurposed drugs selected based on the various filters used in this study are not suitable for SARS-CoV-2 treatment by clinicians and self-medication by people. As a next step, the selected drugs will be evaluated against SARS-CoV-2 and its VOCs in relevant in vitro/ex vivo models, and the results will be communicated elsewhere.

Of 4278 compounds with no assay data, only 453 drugs are FDA/TGA approved. Out of these, 242 compounds were filtered out due to duplication, unsuitable indication, or under clinical evaluation. We recommend the remaining 202 compounds as a priority for generating SARS-CoV-2 assay data. In addition, an interactive and easy to use web-interface (CoviRx) has been developed to provide information regarding these ~7000 compounds. More details regarding this can be found in Jain et al. [20].

## Figures and Tables

**Table 1:** Drug databases used for data curation.

Database	Purpose
Selleckchem epigenetics, Selleck Kinase inhibitors [30] and, MicroSource spectrum libraries [31]	Extraction of drugs for curation
PubChem [32] Drug bank [33], Drug central [34], ChEMBL [35]	Drug identifiers; PK-PD parameters
Clinicaltrials.gov (accessed on 7 February 2022) [36]; WHO clinical trials [37]	Clinical trial status
Drug repurposing hub [38]	Target and mechanism of action
Binding DB [39]	IC_50_ values for original indication
Drugs@FDA [40]	FDA approval status
Australian Register of Therapeutic goods, Prescribing Medicines in Pregnancy Database [41,42]	TGA approval status; pregnancy category
Drugs and lactation database, Australian Register of Therapeutic Goods, Medscape [43,44]	Breastfeeding data
KEGG database [45] and ATC index [46]	Target, category, and indication

**Table 2:** Top 15 drugs that passed all the filters showing their activity rank score against SARS-CoV-2 (with a lower score showing greater activity), original indication, mechanism of action, and associated targets.

Name	Rank Score	Indication	Mechanism of Action	Target
L-cycloserine	0.0098765	Anti-bacterial (tuberculostatic)	-	-
Tolterodine (tartrate)	0.0271605	Urinary anti-spasmodics, Overactive bladder agent	Acetylcholine receptor antagonist (anticholinergic)	CHRM1, CHRM2, CHRM3, CHRM4, CHRM5
Moxidectin	0.0641926	Anti-parasitic	Chloride channel antagonist	
Pyrimethamine	0.0989685	Anti-malarial	Dihydrofolate reductase inhibitor	DHFR, SLC47A1
Meclizine hydrochloride	0.1115153	Anti-emetic	Constitutive androstane receptor (CAR) agonist, Histamine receptor antagonist (antihistamine)	NR1I3
Cysteamine hydrochloride	0.1283852	Anti-urolithic	Tissue transglutaminase inhibitor	NPY2R, SST
Deflazacort	0.153934	Anti-inflammatory	Glucocorticoid receptor agonist	NR3C1
Nifurtimox	0.1808927	Antiprotozoal	DNA inhibitor	HSPD1
Cefaclor	0.1863393	Antibacterial	Bacterial cell wall synthesis inhibitor	
Mianserin hydrochloride	0.1925777	Antidepressant	Serotonin receptor antagonist	ADRA1A, ADRA1B, ADRA1D, HRH1, HRH2, HTR2A, HTR2B, HTR2C, HTR6, HTR7
Procyclidine hydrochloride	0.2006018	Antiparkinsonian, Skeletal muscle relaxant	Acetylcholine receptor antagonist (anticholinergic)	
Palonosetron (hydrochloride)	0.2313175	Anti-emetic	Serotonin receptor antagonist	HTR3A
Gefitinib (ZD1839)	0.2314783	Anti-cancer	Epidermal Growth Factor Receptor (EGFR) inhibitor	EGFR
Dapoxetine	0.2358875	Antidepressant	Selective serotonin reuptake inhibitor (SSRI)	HTR1A, HTR1B, HTR2C, SLC6A4
Rilpivirine	0.2394370	Antiviral	Non-nucleoside reverse transcriptase inhibitor (NNRTI)	NR1I2, SCN10A

CHRM(1–5)—Cholinergic Receptor Muscarinic (1–5); DHFR—Dihydrofolate Reductase; SLC47A1—solute carrier family 47, member 1; NR1I3—Nuclear receptor subfamily 1 group I member 3; NPY2R—Neuropeptide Y Receptor Y2; SST—somatostatin; NR3C1—nuclear receptor subfamily 3 group C member 1; HSPD1—Heat shock protein family D member 1; ADRA1—Alpha-1 adrenergic receptor; HRH(1, 2, 6 and 7)—Human histamine receptor family; EGFR—epidermal growth factor receptor; SLC6A4—solute carrier family 6 member 4; SCN10A—Sodium Voltage-Gated Channel Alpha Subunit 10.

**Table 3:** Drugs with an activity rank score < 0.2 that were reconsidered for repurposing against COVID-19 despite not passing the filter criteria.

Name	Initial Filter Failed	Rank Score
Quinidine Hydrochloride monohydrate	CAD, Toxicity	0.0111111
Everolimus	Toxicity, Same class of drug is in clinical trials	0.0140421
Trihexyphenidyl Hydrochloride	COVID IC_50_ > 10(*) original IC_50_	0.0170512
Sorafenib tosylate	CC_50_ < 10, SI < 10, pregnancy	0.0180246
Rolapitant	SI < 10	0.018665
Idarubicin (Hydrochloride)	CC_50_ < IC_50_, Pregnancy, PAINS, Side effects	0.0342517
Regorafenib (BAY 73-4506)	CC_50_ < 10, SI < 10, pregnancy, Side effects	0.0371304
Itraconazole Hydrochloride	CC_50_ < 10; Same class of drug is in clinical trials, PAINS	0.0411262
Prasterone	Same class of drug in clinical trials	0.0703704
Gemcitabine Hydrochloride	ROA, Pregnancy, Toxicity	0.0712136
Pimecrolimus	ROA, Same class of drug is in clinical trials, Toxicity	0.0742227
Doxepin (Hydrochloride)	Side effects	0.0790123
Abiraterone acetate	COVID IC_50_ > 10(*) original IC_50_, Pregnancy	0.0868782
Cabozantinib (XL184_ BMS-907351)	COVID IC_50_ > 10(*) original IC_50_, Pregnancy, Toxicity	0.0929004
Raloxifene Hydrochloride	CAD, Pregnancy, Toxicity	0.0973509
Avobenzone	Indication	0.1024691
Vinblastine (sulfate)	Pregnancy	0.1109676
Cobimetinib (racemate)	Pregnancy	0.1164189
Zotarolimus	Same class of drug is in clinical trials	0.1578381
Pexidartinib	SI < 10, Side effects	0.1586386
Digoxin	CC_50_ < 10	0.1666667
Thioguanine	CC_50_ < 10, SI < 10	0.1722083
Mebrofenin	Indication	0.1798481
Lenvatinib (E7080)	Pregnancy	0.1801718
Piperonyl butoxide	ROA, Indication	0.181471
Nortriptyline Hydrochloride	PAINS, Side effects	0.1830491
Letermovir	COVID IC_50_ > 10(*) original IC_50_	0.1851185
Thiothixene	PAINS, Side effects	0.1858974

CC50—Cytotoxicity at 50% concentration; IC50 = Inhibitory concentration at 50% concentration; SI = Selectivity Index; ROA = Route of Administration; CAD = Cationic and Amphiphilic drugs; PAINS = Pan Interference Assay; * = folds

**Table 4:** Final prioritisation of drugs for repurposing based on adjusted Cmax calculations.

Drug Name	Cmax (µM)	Protein Binding (%)	Adjusted Cmax Based on Protein Binding (µM)
L-cycloserine	830	“No protein binding”	830
Pyrimethamine	0.94	87	0.1222
Ondansetron	0.43–0.66	73	0.12–0.18
Cyclizine	0.26	60–76 in rats	0.0624
Everolimus	0.186	74	0.04836
Lapatinib	4.18	>99	0.0418
Cetirizine	0.8	93–96	0.032
Rolapitant	1.9	99.8	0.0038
Gefitinib	0.19	90–97	0.0095
Mianserin	0.15	95	0.0075
Palonosetron	0.019	62	0.00722
Meclizine	0.02	99	0.0002

**Table 5:** Sub-filters used for the study.

Filter Type	Description	Objective
Clinical trials (A)	Clinicaltrials.gov database was used to search the drugs under clinical evaluation against SARS-CoV-2 infection. Simultaneously, the Tanimoto index was used to look for the analogues of drugs under clinical trials against SARS-CoV-2 and these were also excluded from our study as a similar scaffold produces similar action.	To prevent duplication of existing work.
CC_50_ < 10 µM Or SI < 10 (B)	Compounds with CC_50_ value < 10µM were considered toxic, while those with >10 µM were deemed non-toxic. Hence, the drugs with CC_50_ values below 10µM were filtered out. In addition, the selectivity index (SI) was also determined, and SI < 10 was considered the minimum acceptable efficacy. *Selectivity index* (*SI*) = *CC*_50_/*IC*_50_	To filter out cytotoxic drugs.
COVID-19 IC_50_ > 10(*) Original Indication (C)	The drug that has ten times more than IC_50_ of original indication are usually toxic, as high doses are needed to show an inhibitory effect.	To filter out drugs that have poor IC_50_ values.
CAD/PAINS (D)	We removed cationic amphiphilic drugs (CAD) that exhibit antiviral activity by inducing phospholipidosis rather than interacting with a specific target. We also removed compound classes that cause pan-assay interference (PAINS) [76].	To screen out false-positive results.
Route of administration (E)	Drugs that are deliverable by oral or inhalation routes were considered in our study, as other routes of administration would limit applicability for the treatment of SARS-CoV-2 infection. Hence, oral and inhalation drugs were retained, and the rest were filtered out.	To filter out drugs that are not orally bioavailable.
Pregnancy (F)	Pregnant women with SARS-CoV-2 infection have been a subject of concern as the present drugs approved for COVID-19 cannot be used to treat them. Hence, drug pregnancy categories were found from the ARTG database and category D and X drugs were removed.	To remove drugs unsafe for use in pregnancy.
Black box warning (G)	Black box warning refers to serious side effects [77]	Filter out drugs with black box warnings to obtain drugs that are safe to use.
Indication (H)	Compounds that have no pharmacological action are also in the database. Hence, all the pharmaceutical aids, diagnostic agents, and supplements were filtered out.	To retain pharmacologically active drugs.

* = folds; ARTG = Australian Register of Therapeutic Goods.

## Data Availability

Not applicable.
